# Genetic and Molecular Insights into the Links Between Heat Stroke, Alzheimer’s Disease, and Down Syndrome: A Mini-Review

**DOI:** 10.3390/genes16101171

**Published:** 2025-10-05

**Authors:** Hisahide Nishio, Hirokuni Negishi, Hiroyuki Awano, Jumpei Oba

**Affiliations:** 1 Department of Occupational Therapy, Faculty of Rehabilitation, Kobe Gakuin University, 518 Arise, Ikawadani-cho, Nishi-ku, Kobe 651-2180, Japan; j-oba@reha.kobegakuin.ac.jp; 2 Social Welfare Corporation AIWAKAI, 1-1-10 Terauchi, Toyonaka 561-0872, Japan; negishi.hirokuni@aijinkai-group.com; 3 Organization for Research Initiative and Promotion, Research Initiative Center, Tottori University, Nishi-cho, Yonago 683-8503, Japan; awano@tottori-u.ac.jp

**Keywords:** heat-related illness, heat stroke, Alzheimer’s disease, down syndrome, down syndrome-related Alzheimer’s disease, down syndrome regression disorder

## Abstract

Both epidemiological and animal model studies have revealed that heat stroke is closely related to the development or exacerbation of dementia disorders. The most common form of dementia is Alzheimer’s disease, which is characterized by the accumulation of amyloid-β protein in the central nervous system. Notably, a whole-genome transcriptome analysis of heat stroke patients has identified the increased expression of amyloid-β precursor protein gene and the activation of amyloid processing pathways. This finding provides a molecular basis for the theory that heat stroke is a risk factor for dementia disorders. Down syndrome—a common chromosomal abnormality—is also a dementia disorder that is characterized by the overexpression of amyloid-β precursor protein gene and the accumulation of amyloid-β protein. Thus, heat stroke may also develop or exacerbate Alzheimer’s disease-like dementia in Down syndrome. For individuals with Down syndrome, heat stroke is therefore not only a life-threatening risk factor but may also be a risk factor for accelerating intellectual decline.

## 1. Introduction

The link between climate change and the frequency and intensity of heat waves is now well established [1]. Global warming threatens our health [2]. This goes beyond heat stroke as an acute illness; heat stroke can also develop or exacerbate neurodegenerative diseases.

Heat stroke has been proposed as a risk factor for neurodegenerative diseases. In 2021, Bongioanni et al. [3] linked the worldwide increase in the number of patients with neurodegenerative diseases to global warming. In 2022, Buizza et al. [4] reported a significant association between climate change and epidemiological data from patients with Parkinson’s disease, noting that the number of patients with Parkinson’s disease increased in countries that experienced severe warming. In 2023, Kuo et al. [5] reported that heat stroke patients are at higher risk of dementia, including Alzheimer’s disease. Although the study did not consider sociodemographic factors such as a family history of dementia, lifestyle habits, and education level, their findings clearly indicate that heat stroke may be a risk factor for dementia.

Down syndrome, a common chromosomal abnormality, exhibits neuropathological findings that are similar to those of Alzheimer’s disease. In addition, many individuals with Down syndrome develop Alzheimer’s disease-like intellectual decline at a relatively young age [6]. We therefore propose that heat stroke may affect the neurological prognosis of individuals with Down syndrome. On the basis of the existing literature, this mini-review examines the relationship between heat stroke, Alzheimer’s disease, and Down syndrome.

## 2. Heat Stroke May Increase the Risk of Alzheimer’s Disease Development

Heat-related illness is a physiological disorder in which the body is unable to adequately dissipate heat, leading to failed thermoregulation [7]. Heat stroke is the most serious heat-related illness [8]. It results in a lack of fluid and salt in the body; this then leads to the dysfunction of multiple organs, including the nervous system. All patients who exhibit clinical manifestations of heat-related illness should be assumed to have heat stroke and should undergo urgent and aggressive cooling treatment [9].

Heat stroke can be classified as “classic” or “exertional” based on the primary heat source that causes the hyperthermia [10]. Hyperthermia in classic heat stroke is primarily caused by exogenous heat from the environment and can occur even in the absence of physical activity, such as during a heatwave. By contrast, the heat source in exertional heat stroke is primarily endogenous heat that is generated by the metabolic system during physical activity. The present mini-review is written in the context of classic heat stroke.

In recent years, a number of reports have noted that heat waves increase the risk of hospitalization for patients with dementia (including those with Alzheimer’s disease) [11,12]. It has also been reported that stronger heat waves increase the mortality rate for patients with dementia [13]. Extra care for patients with dementia is therefore crucial in extreme heat environments.

It should be noted that heat stroke is not merely an acute illness but may also trigger chronic neurological disorders. According to Sinigaglia-Coimbra et al. [14], postischemic hyperthermia in rats induces chronic neuronal cell death in the forebrain. At 2–6 months after recovery, morphological features in these rats are similar to those observed in human Alzheimer’s disease; namely, widely distributed amyloid plaques in the cerebral cortex and the intracellular deposition of hyperphosphorylated tau protein. Taken together, these findings suggest that hyperthermia may trigger Alzheimer’s disease-like pathological conditions and lead to cognitive decline in the long term.

Miyamoto et al. [15] reported that mice exposed to water restriction under high-temperature and -humidity conditions develop delayed neurological damage. These authors observed white matter demyelination and Purkinje cell degeneration in the mice at autopsy and noted that the reduced Purkinje cell numbers did not recover for at least 9 months. These results suggest that heat stroke-induced damage to the central nervous system is permanent and irreversible.

In a cohort study, Kuo et al. [5] reported that heat stroke patients have a higher risk of dementia, including Alzheimer’s disease, than those without heat stroke risk (adjusted hazard ratio = 1.26, 95% confidence interval: 1.18–1.34). Although their epidemiological study had some limitations regarding confounding factors (particularly lifestyle habits), their study still provided us with a significant association between heat stroke and Alzheimer’s disease.

Kuo et al. also conducted animal behavioral tests, noting that rats exposed to a hot and humid environment exhibit cognitive dysfunction even after these conditions disappear [5]. Importantly, significant neuronal damage, degeneration, apoptosis, and amyloid plaque deposition were observed in the hippocampus after heat stroke. These pathological findings of rats exposed to a hot and humid environment suggest that heat stroke may be a risk factor of Alzheimer’s disease, consistent with the study reported by Sinigaglia-Coimbra et al. [14].

## 3. Heat Stroke Increases the Expression of Amyloid-β Protein in Human Brain

Alzheimer’s disease is a leading cause of dementia; it currently affects tens of millions of people worldwide and is projected to continue to increase in the future [16]. Dementia usually occurs in people over 60 years of age, with a lifetime risk of more than 50% [17].

The brains of patients with Alzheimer’s disease exhibit increased extracellular amyloid plaques and intraneuronal neurofibrillary tangles [16]. Symptoms of Alzheimer’s disease correlate with the accumulation of amyloid plaques and neurofibrillary tangles and are a direct result of the damage and destruction of synapses, which control memory and cognition [16].

Interestingly, in Alzheimer’s disease, some stress (including heat stress) triggers a chain reaction of accumulation of amyloid plaques and neurofibrillary tangles. Heat stress increases production of amyloid-β protein and γ-secretase complex formation [18], and then aggregation of amyloid-β protein and formation of amyloid plaques [19].

The amyloid-β aggregation is accompanied by thermogenesis, which also promotes further amyloid-β aggregation and amyloid plaque formation and maturation [20]. Meanwhile, amyloid-β plaques trigger aggregation of phosphorylated tau protein, which increases neurofibrillary tangles [21].

Neuroinflammation triggered by amyloid plaques is now considered to play a pivotal role in the pathogenesis of Alzheimer disease [22]. Hyperthermia induces neuroinflammation due to inflammatory cytokines [23]. Such neuroinflammation induced by hyperthermia may also be associated with pathogenesis of Alzheimer’s disease.

The rapid and generalized progression of symptoms seen in some patients with Alzheimer’s disease is thought to be caused by a prion-like mechanism [24,25], in which toxic aggregates of amyloid-β and tau self-replicate and spread throughout the brain.

Regarding gene expression analysis of heat stroke patients, Bouchama et al. [26] analyzed the whole-genome transcriptome of peripheral blood mononuclear cells from a cohort of subjects exposed to the same high-temperature environment, comparing patients who developed heat stroke (*n* = 19) with those who did not (*n* = 19). The mean rectal temperature of patients who developed heat stroke at admission was 41.7 °C ± 0.8 °C, and eight patients were in a deep coma (Glasgow Coma Score = 3).

Transcriptome analysis revealed that the differentially expressed genes between the two groups encoded proteins related to the unfolded protein response, DNA repair, energy metabolism, oxidative stress, and immunity. Notably, the most highly expressed genes during heat stroke were those encoding proteins that protect the proteome from misfolding and aggregation (e.g., heat shock protein genes) [26]. These results suggest that even an evolutionary heat stress response that includes high heat shock protein gene expression cannot completely prevent heat injury.

Bouchama et al. [26] also reported that patients with heat stroke showed increased expression of the amyloid-β precursor protein gene (*APP*) as well as genes in the amyloid processing pathway [20]. These findings suggested that heat stroke may develop or exacerbate Alzheimer’s disease and are consistent with the epidemiological data from Kuo et al. [5]. However, the whole-genome transcriptome analysis by Bouchama et al. [26] was limited by the sample size, and there were concerns about whether the pathology of peripheral blood mononuclear cells faithfully reflected the pathology of neurons.

**Figure 1:**
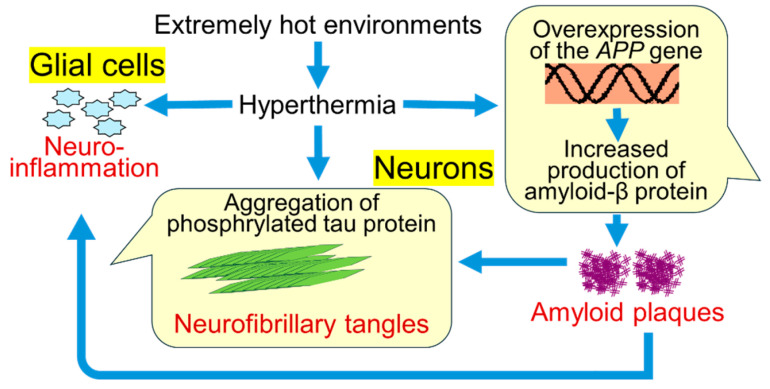
**Hyperthermia-related pathways to development or exacerbation of Alzheimer’s disease**. Hyperthermia induces overexpression of the APP gene, leading to an increase in amyloid-β protein. Increased amyloid-β protein facilitates amyloid plaque formation outside neurons. Accumulation of amyloid plaques synergizes aggregation of phosphorylated tau protein (or neurofibrillary tangle formation) and promotes neuroinflammation. Hyperthermia also stimulates neuroinflammation and aggregation of tau protein. By a similar mechanism, in individuals with Down syndrome, hyperthermia may activate the pathways to development or exacerbation of Alzheimer’s disease.

Figure 1 is a summary of previous studies on the relationships between hyperthermia and development or exacerbation of Alzheimer’s disease.

## 4. What Effect Does Heat Stroke Have on Individuals with Down Syndrome?

Down syndrome is caused by three copies of chromosome 21 and represents the most common chromosomal disorder in humans. Its primary symptoms include developmental disorders in childhood and dementia in adulthood [6]. Individuals with Down syndrome often have underlying issues with the autonomic nervous system, leading to inadequate thermoregulation and poor surface blood circulation [27,28,29]. A Down syndrome support website also notes that individuals with Down syndrome tend to sweat less [30], which may mean that individuals with Down syndrome are vulnerable to heat.

Heat stroke as an acute illness can be life-threatening, and preventative measures must therefore be implemented. However, individuals with Down syndrome may have difficulty recognizing and expressing their own discomfort or pain [31,32,33]. This means that, without close monitoring by caregivers, heat stroke in these individuals can rapidly progress, leading to severe fluid and salt imbalances and even death. These issues represent the risks of heat stroke as an acute illness.

We believe that heat stroke may also be related to the long-term neurological prognosis of Down syndrome. Overexpression of the *APP gene*, located on chromosome 21, has been observed in the brains of individuals with Down syndrome, and Down syndrome progresses in a manner similar to that of Alzheimer’s disease [34]. In the brains of individuals with Down syndrome, amyloid plaques and tau neurofibrillary tangles occur almost universally by age 40, and the lifetime risk of developing dementia exceeds 90% [35]. Moreover, in Down syndrome—as in Alzheimer’s disease—amyloid-β and tau contribute to neurodegeneration [36].

In addition to amyloid plaques and tau neurofibrillary tangles, pathological changes in Down syndrome include abnormal oligodendrocyte differentiation and hypomyelination. Olmos-Serrano et al. [37] conducted a multi-region transcriptome analysis of the brains of individuals with Down syndrome and controls from mid-fetal development to adulthood. The results revealed genome-wide changes in the expression of numerous genes. These authors also identified the dysregulation of genes related to oligodendrocyte differentiation and myelination [37]. Nonetheless, further investigation is needed to determine the relationships between amyloid/tau deposition and defects in oligodendrocyte differentiation and myelination.

Nearly all individuals with Down syndrome develop Alzheimer’s disease-like neuropathology by 40 years of age, as mentioned above, and Alzheimer’s disease-like dementia tends to appear after 40 years of age [6]. Approximately 80% of all individuals with Down syndrome aged 60–69 years, and up to 100% of those aged 70 years or older, present with Alzheimer’s disease-like dementia (also known as Down syndrome-related Alzheimer’s disease) [6]. On the other hand, in typical Alzheimer’s disease, dementia usually occurs in individuals over 60 years of age, with a lifetime risk of more than 50% [17].

Recently, there have also been many reports of subacute neurocognitive regression in individuals with Down syndrome [38]. This clinical condition is also known as Down syndrome regression disorder (DSRD), Down syndrome disintegrative disorder, Down syndrome regression of unknown cause, or idiopathic regression in Down syndrome [38,39]. Herein, we use the term DSRD, which refers to the rapid loss of previously acquired developmental skills in areas such as language, communication, cognition, executive function, behavior, and adaptive skills [38].

Walpert et al. [39] argued that DSRD differs from Alzheimer’s disease because the symptoms in DSRD may halt or improve, whereas those in Alzheimer’s disease continue to progress. A scoping review by Natividade et al. [40] noted that DSRD is usually observed at around the age of 20 years and stated that the causes (or contributing factors) may be antecedent psychosocial stress or autoimmune disease. Worley et al. [41] reported that 10 of 11 patients with DSRD had elevated thyroid peroxidase antibody titers. This finding suggests that autoimmune hypothyroidism (or Hashimoto’s disease) may be a cause of rapid regression in Down syndrome; autoimmunity appears to be substantially involved in DSRD.

DSRD may have multiple causes. In daily clinical practice, we have observed patients with DSRD who do not have Hashimoto’s disease. Furthermore, the possibility that DSRD is Down syndrome with very early onset of Alzheimer’s disease cannot be ruled out. Nevertheless, we hypothesize that, just as heat stroke is a risk factor for Alzheimer’s disease, heat stroke may be an exacerbating factor for Down syndrome or a promoting factor for DSRD.

## 5. Conclusions

In this review article, we explained the relationship between heat stroke and Alzheimer’s disease, citing several literature sources. Here, it was emphasized that heat stroke is a risk factor for Alzheimer’s disease. However, hyperthermia also induces heat shock proteins, which help prevent brain damage.

A whole-genome transcriptome analysis of heat stroke patients revealed the elevated expression of genes encoding heat shock proteins [20]. Heat shock proteins undoubtedly provide some protection against the health hazards of high-temperature environments; however, heat stroke remains a risk factor for the future development of Alzheimer’s disease [5]. That is, even the evolutionary heat stress response—which includes elevated heat shock protein gene expression—cannot completely prevent heat damage leading to Alzheimer’s disease.

Here, we also suggested that hyperthermia may lead to a long-term decline in intellectual ability, particularly in individuals with Down syndrome. Although no full-scale cohort studies have been conducted in individuals with Down syndrome, it is almost certain that heat stroke is not only a life-threatening acute illness in individuals with Down syndrome, but also a risk factor for the early development of Alzheimer’s disease.

We therefore conclude that (1) heat stroke is a risk factor for Alzheimer’s disease, and (2) heat stroke may accelerate the early onset or rapid regression of Down syndrome-related Alzheimer’s disease in individuals with Down syndrome.

Regarding the limitations of our study, this review article does not cover impaired physiological functions including vascular damage, systemic inflammation, or metabolic dysfunction. In our next review article, cellular gene expression and the biological responses of individual organs will be discussed comprehensively.

Finally, we hope that future epidemiological studies will include longitudinal studies of heat-exposed patients with Down syndrome, and experimental studies will explore the molecular mechanisms of heat stroke using iPSC-derived neurons or organoids.

We also hope that further understanding of the pathogenesis of subacute neurocognitive regression in Down syndrome (i.e., DSRD) will shed light on the potential risk of heat stroke in this pathological mechanism. We are currently conducting clinical research to elucidate the pathogenesis of subacute neurocognitive regression in Down syndrome and to clarify the potential role of heat stroke in this pathological mechanism.

## Data Availability

No new data were created or analyzed in this study. Data sharing is not applicable to this article.

## References

[1] Luber G., McGeehin M. (2008). Climate change and extreme heat events. Am. J. Prev. Med..

[2] Zhao Q., Guo Y., Ye T., Gasparrini A., Tong S., Overcenco A., Urban A., Schneider A., Entezari A., Vicedo-Cabrera A.M. (2021). Global, regional, and national burden of mortality associated with non-optimal ambient temperatures from 2000 to 2019: A three-stage modelling study. Lancet Planet Health.

[3] Bongioanni P., Del Carratore R., Corbianco S., Diana A., Cavallini G., Masciandaro S.M., Dini M., Buizza R. (2021). Climate change and neurodegenerative diseases. Environ. Res..

[4] Buizza R., Del Carratore R., Paolo Bongioanni P. (2022). Evidence of climate change impact on Parkinson’s disease. J. Clim. Change Health.

[5] Kuo W.Y., Huang C.C., Chen C.A., Ho C.H., Tang L.Y., Lin H.J., Su S.B., Wang J.J., Hsu C.C., Chang C.P. (2024). Heat-related illness and dementia: A study integrating epidemiological and experimental evidence. Alzheimers Res. Ther..

[6] Antonarakis S.E., Skotko B.G., Rafii M.S., Strydom A., Pape S.E., Bianchi D.W., Sherman S.L., Reeves R.H. (2020). Down syndrome. Nat. Rev. Dis. Primers.

[7] Gauer R., Meyers B.K. (2019). Heat-related illnesses. Am. Fam. Physician.

[8] Bouchama A., Knochel J.P. (2002). Heat stroke. N. Engl. J. Med..

[9] O’Connor F.G. (2025). Heat-related illnesses. Ann. Intern. Med..

[10] Bouchama A., Abuyassin B., Lehe C., Laitano O., Jay O., O’Connor F.G., Leon L.R. (2022). Classic and exertional heat stroke. Nat. Rev. Dis. Primers.

[11] Xu Z., Tong S., Cheng J., Zhang Y., Wang N., Zhang Y., Hayixibayi A., Hu W. (2019). Heatwaves, hospitalizations for Alzheimer’s disease, and postdischarge deaths: A population-based cohort study. Environ. Res..

[12] Gong J., Part C., Hajat S. (2022). Current and future burdens of heat-related dementia hospital admissions in England. Environ. Int..

[13] Zhang R., Sun L., Jia A., Wang S., Guo Q., Wang Y., Wang C., Wu S., Zheng H., Su X. (2024). Effect of heatwaves on mortality of Alzheimer’s disease and other dementias among elderly aged 60 years and above in China, 2013-2020: A population-based study. Lancet Reg. Health West. Pac..

[14] Sinigaglia-Coimbra R., Cavalheiro E.A., Coimbra C.G. (2002). Postischemic hyperthermia induces Alzheimer-like pathology in the rat brain. Acta Neuropathol..

[15] Miyamoto K., Nakamura M., Ohtaki H., Suzuki K., Yamaga H., Yanagisawa K., Maeda A., Yagi M., Hayashi M., Honda K. (2022). Heat stroke-induced late-onset neurological deficits in mice caused by white matter demyelination, Purkinje cell degeneration, and synaptic impairment in the cerebellum. Sci. Rep..

[16] Hampel H., Hardy J., Blennow K., Chen C., Perry G., Kim S.H., Villemagne V.L., Aisen P., Vendruscolo M., Iwatsubo T. (2021). The amyloid-β pathway in Alzheimer’s disease. Mol. Psychiatry.

[17] Yoshida D., Ohara T., Hata J., Shibata M., Hirakawa Y., Honda T., Furuta Y., Oishi E., Sakata S., Kanba S. (2020). Lifetime cumulative incidence of dementia in a community-dwelling elderly population in Japan. Neurology.

[18] Noorani A.A., Yamashita H., Gao Y., Islam S., Sun Y., Nakamura T., Enomoto H., Zou K., Michikawa M. (2020). High temperature promotes amyloid β-protein production and γ-secretase complex formation via Hsp90. J. Biol. Chem..

[19] Chandhok S., Pereira L., Momchilova E.A., Marijan D., Zapf R., Lacroix E., Kaur A., Keymanesh S., Krieger C., Audas T.E. (2023). Stress-mediated aggregation of disease-associated proteins in amyloid bodies. Sci. Rep..

[20] Chung C.W., Stephens A.D., Konno T., Ward E., Avezov E., Kaminski C.F., Hassanali A.A., Kaminski Schierle G.S. (2022). Intracellular Aβ42 Aggregation Leads to Cellular Thermogenesis. J. Am. Chem. Soc..

[21] Busche M.A., Hyman B.T. (2020). Synergy between amyloid-β and tau in Alzheimer’s disease. Nat. Neurosci..

[22] Heneka M.T., van der Flier W.M., Jessen F., Hoozemanns J., Thal D.R., Boche D., Brosseron F., Teunissen C., Zetterberg H., Jacobs A.H. (2025). Neuroinflammation in Alzheimer disease. Nat. Rev. Immunol..

[23] Yoneda K., Hosomi S., Ito H., Togami Y., Oda S., Matsumoto H., Shimazaki J., Ogura H., Oda J. (2024). How can heatstroke damage the brain? A mini review. Front. Neurosci..

[24] Schmidt C., Wolff M., Weitz M., Bartlau T., Korth C., Zerr I. (2011). Rapidly progressive Alzheimer disease. Arch. Neurol..

[25] Bloom G.S. (2014). Amyloid-β and tau: The trigger and bullet in Alzheimer disease pathogenesis. JAMA Neurol..

[26] Bouchama A., Rashid M., Malik S.S., Al Mahri S., Yassin Y., Abdullah M., Abdulmalek N., Maashi F., Mashi A., Khan A. (2023). Whole genome transcriptomic reveals heat stroke molecular signatures in humans. J. Physiol..

[27] Sinton J.W., Cooper D.S., Wiley S. (2022). Down syndrome and the autonomic nervous system, an educational review for the anesthesiologist. Paediatr. Anaesth..

[28] Surma V., Nagraj V.P., Fairchild K.D., Vergales J. (2020). Temperature instability in infants with trisomy 21 in the neonatal intensive care unit. J. Perinatol..

[29] Dębiec-Bąk A., Wójtowicz D., Pawik Ł., Ptak A., Skrzek A. (2019). Analysis of body surface temperatures in people with Down syndrome after general rehabilitation exercise. J. Therm. Anal. Calorim..

[30] Down Syndrome Resource Foundation Staying Safe in Extreme Heat. https://www.dsrf.org/resources/information/down-syndrome-climate-change/staying-safe-in-extreme-heat/.

[31] McGuire B.E., Defrin R. (2015). Pain perception in people with Down syndrome: A synthesis of clinical and experimental research. Front. Behav. Neurosci..

[32] Hennequin M., Morin C., Feine J.S. (2000). Pain expression and stimulus localisation in individuals with Down’s syndrome. Lancet.

[33] Abu-Saad H.H. (2000). Challenge of pain in the cognitively impaired. Lancet.

[34] Maure-Blesa L., Carmona-Iragui M., Lott I., Head E., Wisniewski T., Rafii M.S., Espinosa J., Flórez J., Mobley W.C., Holland A. (2025). The history of Down syndrome-associated Alzheimer’s disease; past, present, and future. Alzheimers Dement..

[35] Fortea J., Zaman S.H., Hartley S., Rafii M.S., Head E., Carmona-Iragui M. (2021). Alzheimer’s disease associated with Down syndrome: A genetic form of dementia. Lancet Neurol..

[36] Condello C., Maxwell A.M., Castillo E., Aoyagi A., Graff C., Ingelsson M., Lannfelt L., Bird T.D., Keene C.D., Seeley W.W. (2022). Aβ and tau prions feature in the neuropathogenesis of Down syndrome. Proc. Natl. Acad. Sci. USA.

[37] Olmos-Serrano J.L., Kang H.J., Tyler W.A., Silbereis J.C., Cheng F., Zhu Y., Pletikos M., Jankovic-Rapan L., Cramer N.P., Galdzicki Z. (2016). Down syndrome developmental brain transcriptome reveals defective oligodendrocyte differentiation and myelination. Neuron.

[38] Santoro J.D., Patel L., Kammeyer R., Filipink R.A., Gombolay G.Y., Cardinale K.M., Real de Asua D., Zaman S., Santoro S.L., Marzouk S.M. (2022). Assessment and diagnosis of Down syndrome regression disorder: International expert consensus. Front. Neurol..

[39] Walpert M., Zaman S., Holland A. (2021). A systematic review of unexplained early regression in adolescents and adults with Down syndrome. Brain Sci..

[40] Natividade M.M.P., Moreira A.J., Nassif L.S., Dos Santos B.R.A., Borin M.C., Alvares-Teodoro J., Acurcio F.A., Guerra A.A. (2025). Causes of Down syndrome regression disorder: A scoping review. Dement. Neuropsychol..

[41] Worley G., Crissman B.G., Cadogan E., Milleson C., Adkins D.W., Kishnani P.S. (2015). Down syndrome disintegrative disorder: New-onset autistic regression, dementia, and insomnia in older children and adolescents with Down syndrome. J. Child Neurol..

